# Tunable High-Performance Microwave Absorption and Shielding by Three Constituent Phases Between rGO and Fe_3_O_4_@SiO_2_ Nanochains

**DOI:** 10.3389/fchem.2019.00711

**Published:** 2019-11-26

**Authors:** Chao-Qin Li, Wei Xu, Ruo-Cheng Ding, Xun Shen, Zhi Chen, Mao-Dong Li, Guang-Sheng Wang

**Affiliations:** ^1^Engineering Research Center of High-Performance Polymer and Molding Technology, Ministry of Education, Qingdao University of Science and Technology, Qingdao, China; ^2^School of Chemistry, Beihang University, Beijing, China; ^3^Guangzhou Special Pressure Equipment Testing and Research Institute, Guangzhou, China

**Keywords:** Fe_3_O_4_@SiO_2_ nanochains, rGO/Fe_3_O_4_@SiO_2_ composite, microwave absorption, electromagnetic shielding, multi-functional composite

## Abstract

With the aim of achieving high microwave absorption and electromagnetic shielding performance, reduced graphene oxide (rGO) and Fe_3_O_4_@SiO_2_ nanochains are successfully combined at various mass ratios. By selecting the right mass ratio, an rGO/Fe_3_O_4_@SiO_2_ composite with excellent microwave absorption properties is obtained, and, due to the addition of highly conductive rGO, the desired shielding effectiveness is also achieved. The reflection loss (RL) value of the composite can reach −48.34 dB with a mass ratio of 1:1, and the effective bandwidth (<-10 dB) can cover 4.88 GHz at a thickness of 2.0 mm. Moreover, the composite with a mass ratio of 4:1 exhibits outstanding electromagnetic shielding performance, which also broadens its fields of application. This outstanding microwave absorption and electromagnetic shielding performance indicate that the composite can potentially be employed as a multi-functional material.

**Graphical Abstract:**
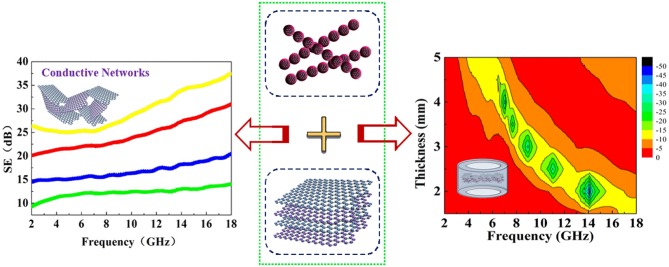
Multi-functional composite with high-performance microwave absorption and shielding based on rGO/Fe_3_O_4_@SiO_2_.

## Introduction

With the wide application of electromagnetic (EM) waves in the GHz frequency band in recent years, electromagnetic pollution has become quite a serious and universal problem (Li et al., 2006; Saini and Arora, 2013). Materials possessing microwave absorption and multiple shielding functions have attracted much attention for their important role not only in attenuating redundant EM energy but in serving as a shield against the entrance of the unwanted EM irradiation. Therefore, great efforts have been directed toward developing various systems to acquire ideal composites, such as the ferrite (Abbas et al., 2007; Zhang et al., 2014), conjugated polymer (Saini et al., 2011), carbonaceous (Liang et al., 2009), or hybrid systems (Singh et al., 2013).

Though it is a typical ferrite material that is low-cost for industrial production, Fe_3_O_4_ is seriously limited in terms of electromagnetic wave absorption and shielding properties. Its microwave absorption performance is usually poorer in the high-frequency range due to the eddy current effect. Focusing on the fabrication of one dimensional (1D) nanomaterials is a useful way to conquer the problem of low permeability values in the high-frequency range on account of Snoek's limit, potentially providing greatly enhanced electromagnetic properties (Chai et al., 2008). The advantages of 1D nanomaterials used as microwave absorbers have already been confirmed by many groups (Qiao et al., 2009; Xiang et al., 2014; Liu et al., 2015; Xu et al., 2018a).

In addition, as a recessive shielding material, ferrite also needs to be made part of a hybrid system, with the aim of overcoming its disadvantages of low conductivity, high density, and corrosion susceptibility (Saini et al., 2013). As the thinnest material in the carbon family, reduced graphene oxide (rGO) has an extremely high specific surface area and carrier mobility, as well as abundant defects and hydroxyl, epoxy, and carboxyl groups (Kim et al., 2014; Guo et al., 2016; Ran et al., 2017). On the basis of previous research, we may also deduce that the hierarchical structure and synergistic effect of rGO-based nanocomposites can balance the complex permittivity and permeability for impedance matching (Yan et al., 2014; Zhang et al., 2017; Xu et al., 2018b). As a result, rGO, as a representative carbonaceous material, is used as a component in multiple applications in microwave absorption and EMI shielding.

In this context, adjusting the ratio of 1D Fe_3_O_4_@SiO_2_ nanochains and rGO is a favorable way to realize permittivity regulation so that the desired properties of electromagnetic wave absorption and shielding can be achieved. The corresponding mechanisms based on complex permittivity, the impedance matching condition, and conductivity are also investigated in detail in the measured frequency range of 2–18 GHz.

## Experimental Methods

### Preparation of Fe_3_O_4_ Magnetic Nanoparticle Clusters (MNCs)

The standard procedure was used, in which a certain proportion of chemicals were sufficiently dissolved in ethylene glycol (Pan et al., 2015), and then the homogeneous solution obtained was heated at 200°C for 10 h through the solvothermal method. Finally, the black precipitates were collected, washed with ethanol and distilled water several times, and then dried in the oven at 60°C.

### Preparation of Fe_3_O_4_@SiO_2_ Nanochains

In a typical synthesis of nanochains, MNCs (0.0258 g) was dispersed in a mixture of distilled water (6 mL), NH_4_OH (1 mL), and ethanol (40 mL) under sonication. The TEOS (120 mL) was then gradually injected into the mixture while stirring. After 20 min, 20 mL of the mixture was moved to a beaker (40 mL) and placed on a 75 × 24 mm disc magnet for 3 s. The sample was then allowed to sit in a non-magnetic field for another 25 min and then washed with ethanol and distilled water several times.

### Preparation of rGO/Fe_3_O_4_@SiO_2_ Composite

First, graphite oxide was synthesized by a modified Hummers method (Hummers and Offeman, 1958). The subsequent rGO preparation was similar to that previously reported by Wang and co-workers (Zhang et al., 2014). A certain amount of nanochains were added to an rGO suspension and subjected to ultrasonic treatment for 2 h. Afterward, the product was separated by centrifugation, washed with ethanol, and freeze-dried at −50°C for 48 h.

### Characterization

XRD patterns were recorded by using an X-ray diffractometer (D/MAX-1200, Rigaku Denki Co. Ltd, Japan) at λ = 0.15406 nm. SEM images were acquired with a Quanta 250 FEG to examine the grain morphology and size. The magnetic curves of the samples were obtained at room temperature by vibrating sample magnetometer (VSM, Riken Denshi Co. Ltd, Japan).

### Measurement of Electromagnetic Parameters

The composites used for EM absorption and shielding measurement were mixed with poly(-vinylidene fluoride) (PVDF) at 2.5 and 20 wt% loading, respectively, and then pressed into compact cylinders (ϕ_*out*_ = 7.00 mm and ϕ_*in*_ = 3.04 mm). The EM parameters were measured by the transmission/reflection coaxial line method in the 2–18 GHz frequency range with an Agilent E5071C Network Analyzer, with the input power level set to −5 dBm.

## Results and Discussion

**Figure 1:**
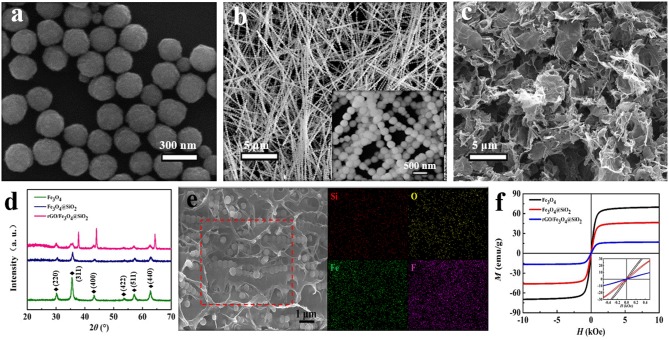
SEM images of **(a)** Fe_3_O_4_ MNCs, **(b)** Fe_3_O_4_@SiO_2_ nanochains, **(c)** rGO/Fe_3_O_4_@SiO_2_, and corresponding **(d)** XRD patterns and **(f)** hysteresis loops at room temperature, **(e)** FESEM image of the fracture surface of rGO/Fe_3_O_4_@SiO_2_/PVDF and corresponding elemental mapping images of Si, O, Fe, and F.

Due to the magnetic interaction, Fe_3_O_4_ MNCs show a certain agglomeration, with a diameter of about 250 nm, as shown in Figure 1a. The magnetic response characteristic and the fixed effect of the hydrolysis product of TEOS also enable the formation of Fe_3_O_4_@SiO_2_ with a one-dimensional chain structure, as shown in Figure 1b, consequently improving the directional transmission function between particles and affecting the ultimate electromagnetic properties. Figures S1a–c shows the morphology of Fe_3_O_4_@SiO_2_ nanochains obtained with different amounts of TEOs. In additional, we have found that controlling the time of the applied magnetic field is a good way to adjust the distance between the nanoparticles in the chain. As shown in Figure S2, when a magnetic field is applied only 5 min after the TEOS is added, there is only a viscous layer of SiO_2_ between the Fe_3_O_4_ MNCs, at which point the SiO_2_ connects the two nanoparticles. While Fe_3_O_4_ MNC was not completely coated with SiO_2_, a multi-stage structure with a rough surface was observed. As the time of adding the magnetic field increases gradually, the SiO_2_ layer encapsulating the nano-chain becomes thicker and thicker, and the distance between the Fe_3_O_4_ MNCs gradually increases. Another key factor in the preparation of uniform nanochains is the uniform magnetic field strength. As shown in Figure S3, an ordered linear chain structure cannot be formed under a non-uniform rectangular magnetic field. Therefore, in this study we used a central portion of a large diameter disc magnet to provide a uniform magnetic field. Figure 1d shows the XRD diffraction patterns of Fe_3_O_4_ MNCs, Fe_3_O_4_@SiO_2_ nanochains, and rGO/Fe_3_O_4_@SiO_2_ composites. The Fe_3_O_4_ MNCs exhibit high crystallinity, and their XRD peaks correspond to the (220), (311), (400), (422), (511), (440) crystal planes, in good agreement with the Fe_3_O_4_ standard card (JSPDF NO. 01-1111). TEOS hydrolysate is an amorphous SiO_2_ material, so there is no diffraction peak of SiO_2_, while Fe_3_O_4_ can maintain a stable chemical state. The XRD results for rGO/Fe_3_O_4_@SiO_2_ composites also show that GO can be effectively reduced to rGO by chemical reduction. As a type of ferro-magnetic ferrite, the magnetic moment of adjacent atoms in Fe_3_O_4_ is antiparallel, showing a certain degree of macroscopic magnetic effect. In addition, under the action of an external magnetic field, Fe_3_O_4_ can generate significant magnetization, resulting in the enhancement of the internal synthetic magnetic field; this is closely related to the electromagnetic performance of composites. As shown in Figure 1f, compared with pure Fe_3_O_4_ MNCs, the saturation magnetization and coercivity values of nanochains and composites, depicted in Figure 1f, are reduced and increased gradually, respectively, mainly due to the addition of amorphous silica and rGO. In additional, the hysteresis loops of Fe_3_O_4_@SiO_2_ nanochains with different amounts of TEOs are shown in Figure S1d. It can be found that the amount of TEOs has a strong influence on the saturation magnetization of Fe_3_O_4_@SiO_2_ nanochains.

**Figure 2:**
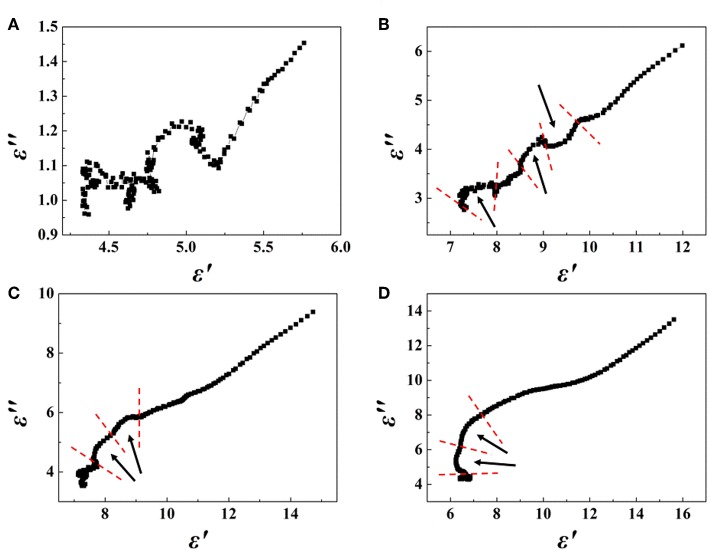
Plots of ε′ vs. ε″ for rGO/Fe_3_O_4_@SiO_2_ composites with **(A)** 1:2, **(B)** 1:1, **(C)** 2:1, **(D)** 4:1 mass ratios.

The complex permittivity (ε_*r*_ = ε′–* j*ε″), complex permeability (μ_*r*_ = μ′–* j*μ″), dielectric loss and magnetic loss of composites are depicted in Figure S1. The real permittivity (ε′) and real permeability (μ′) are related to the storage capacity of EM energy, whereas the imaginary permittivity (ε″) and imaginary permeability (μ″) are connected with the energy dissipation, respectively. Figure S1 shows that the values of ε′ and ε″ tend to rise with an increase in the rGO mass ratio, suggesting that the high conductivity of rGO, which is rich in defects, leads to enhanced conductivity loss and polarization loss, especially in the relatively low frequency range. Moreover, the enhancement of dielectric loss is mainly a result of dipole-oriented polarization and interfacial polarization. In rGO/Fe_3_O_4_@SiO_2_ composites, multiple interfaces occur between rGO, Fe_3_O_4_, SiO_2_, and air cavities, which favors the enhancement of absorption. Besides, the one-dimensional nanochains contribute to expanding the special surface area due to causing more interfacial polarization and scattering of the incident electromagnetic waves. Meanwhile, the rGO nanosheets tend to provide more active sites, which leads to multiple reflections and scattering, dissipating electromagnetic energy by extending the transmission path. In addition, as shown in Figure 2, Debye dipolar relaxation is a useful mechanism by which a dielectric loss material can absorb microwaves. On the basis of the Debye theory, ε′ and ε″ can be expressed as (Wen et al., 2014; Cao et al., 2018):

$$\begin{array}{l} \varepsilon^{\prime }=\varepsilon_{\infty }-\frac{\varepsilon_{s}-\varepsilon_{\infty }}{1+{\left(2\pi f\right)}^{2}\tau^{2}}\\ {\;\varepsilon }^{\prime \prime }=\frac{2\pi f\tau \left(\varepsilon_{s}-\varepsilon_{\infty }\right)}{1+{\left(2\pi f\right)}^{2}\tau^{2}} \end{array}$$

where *f* , ε_*s*_, ε_∞_, and τ are frequency, static permittivity, relative dielectric permittivity at the high frequency limit, and polarization relaxation time, respectively.

According to the above-mentioned expressions, their relationship can be deduced as (Zhang et al., 2018):

$$\begin{array}{l} {\left(\varepsilon^{\prime }-\frac{\varepsilon_{s}-\varepsilon_{\infty }}{2}\right)}^{2}+{\left(\varepsilon^{\prime \prime }\right)}^{2}={\left(\frac{\varepsilon_{s}-\varepsilon_{\infty }}{2}\right)}^{2} \end{array}$$

Thus, the curve of ε′ vs. ε″ would be a single semicircle, generally denoted as the Cole–Cole semicircle (Pan et al., 2017). An effective Debye dipolar relaxation process regularizes the semicircular shape.

In addition to dielectric loss, magnetic loss is mainly derived from hysteresis, domain wall resonance, natural resonance, and the eddy current effect. However, the hysteresis loss can be ignored in a weak field, and domain wall resonance loss always appears at MHz frequencies. Therefore, the attenuation of EM waves may be attributed to the eddy current effect and natural resonance. The eddy current loss can be computed by the following formula (Wu et al., 2016):

$$\begin{array}{l} \mu^{\prime \prime }\approx 2\pi \mu_{0}{\left(\mu^{\prime }\right)}^{2}\sigma d^{2}f/3 \end{array}$$

where σ (S cm^−1^) is the electrical conductivity and μ_0_ (H m^−1^) is the permeability. The values of *C*_0_ (*C*_0_ = μ″ *(*μ′*)*^−2^*f*^−1^) remain constant when the frequency varies, leading to the reflection loss caused by the eddy current loss effect. It can be observed from Figure S2a that the values of μ″ *(*μ′*)*^−2^*f*^−1^ hardly fluctuate within the frequency range from 6 to 18 GHz at a mass ratio of 4:1, implying that eddy current loss plays a role. The natural resonance, meanwhile, can be described by the following formula (Jian et al., 2016):

$$\begin{array}{l} H_{a}=4|K_{1}|/3\mu_{0}M_{S} \end{array}$$

where |*K*_1_| is the anisotropic coefficient and *Ms* is the saturation magnetization. Depending on the effective anisotropy field, the resonance frequency is linked with the coercivity values of the materials. When the *Ms* value of the rGO/Fe_3_O_4_@SiO_2_ is low, the rGO/Fe_3_O_4_@SiO_2_ composites possess higher anisotropic energy, benefitting their microwave absorption performance.

Moreover, with regard to attenuation properties, the attenuation constant α can be expressed as (Liu et al., 2010):

$$\begin{array}{l} \alpha =\frac{\sqrt{2}\text{π}f}{c}\times \\ \sqrt{\left(\mu^{\prime \prime }\varepsilon^{\prime \prime }-\mu^{\prime }\varepsilon^{\prime }\right)+\sqrt{{\left(\mu^{\prime \prime }\varepsilon^{\prime \prime }-\mu^{\prime }\varepsilon^{\prime }\right)}^{2}+{\left(\mu^{\prime }\varepsilon^{\prime \prime }+\mu^{\prime \prime }\varepsilon^{\prime }\right)}^{2}}} \end{array}$$

where *f* is the frequency and *c* is the velocity of light in a vacuum. As shown in Figure S2b, the attenuation constant of the composite, similarly to the dielectric constant (Figure S1), increases with a larger proportion of rGO. According to the balance between the impedance matching ratio and attenuation constant, it can be deduced that a mass ratio of 2:1 or 1:1 in the composites may be desirable.

The above conclusion is verified by calculating on the basis of the measured data of the complex permittivity and complex permeability, presented in Supplementary Material, the reflection loss (RL) values can be calculated according to the transmission line theory, which is summarized by the following equations (Cao et al., 2010):

$$\begin{array}{l} \;R=20\log\left|\frac{Z_{in}-1}{Z_{in}+1}\right|\\ Z_{in}=\sqrt{\frac{\mu_{r}}{\varepsilon_{r}}}\tanh\left[j\left(\frac{2f\pi d}{c}\right)\sqrt{\mu_{r}\varepsilon_{r}}\right] \end{array}$$

where *Z*_*in*_ is the normalized input characteristic impedance, ε_*r*_ and μ_*r*_ are the complex permittivity and permeability of the composite absorber, respectively, *d* is the thickness of the absorber, *f* is the frequency, and *c* is the velocity of light in free space.

**Figure 3:**
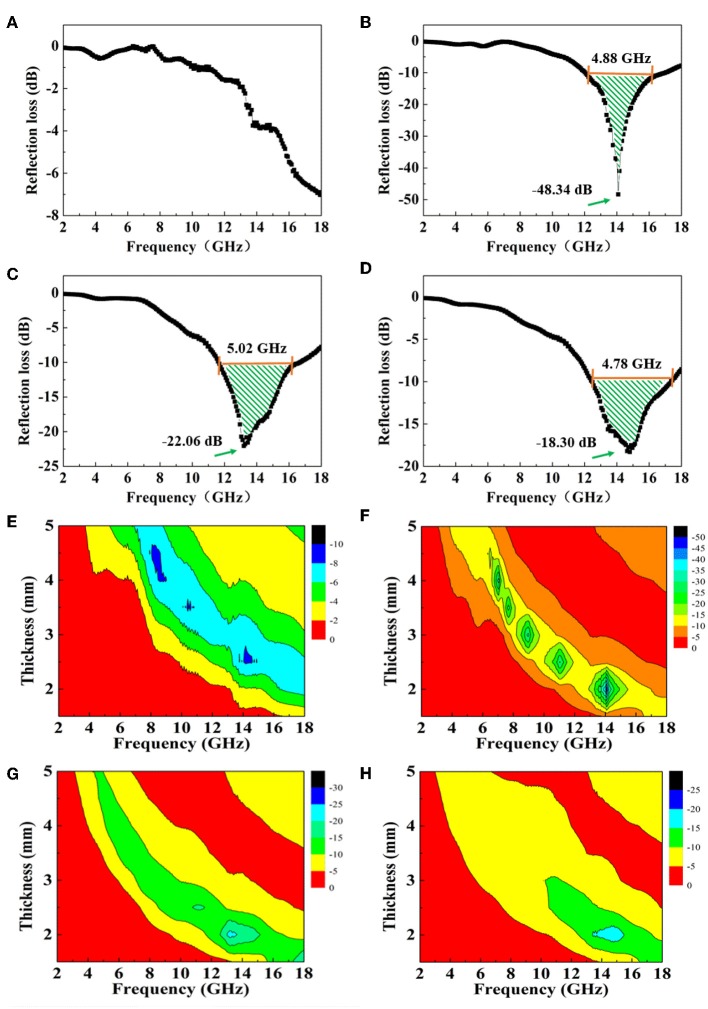
RL curves for rGO/Fe_3_O_4_@SiO_2_ composites with **(A)** 1:2, **(B)** 1:1, **(C)** 2:1, **(D)** 4:1 mass ratios at a thickness of 2.0 mm, and corresponding color fill versions of RL **(E–H)** in the range 1.5–5.0 mm.

The RL curves of rGO/Fe_3_O_4_@SiO_2_ composites with different mass ratios at a thickness of 2.0 mm are displayed in Figure 3. Generally, 90% of irradiated waves would be absorbed when the RL value reaches −10 dB, and 99% irradiation absorption likewise corresponds to −20 dB. It can be found that the electromagnetic wave absorption peak of the composite first increases and then decreases as the mass ratio of rGO rises. When the mass ratio of rGO/Fe_3_O_4_@SiO_2_ is 1:1, the maximum absorption peak of the composite reaches −48.34 dB, and the effective absorption bandwidth covers 4.88 GHz. When the mass ratio is 2:1, the effective absorption bandwidth reaches 5.02 GHz, indicating that it can be used as a broad bandwidth absorbing agent. An effective wave absorber shows not only high absorption intensity but also a wide absorption band, low filler content, and thinness. The electromagnetic wave absorption performance of Fe_3_O_4_@SiO_2_ nanochains is compared in Figure S7 for illustrating the enhancement of absorbing properties of composites by rGO. What's more, in order to more fully represent the absorbing properties of the composites, the RL curves of rGO/Fe_3_O_4_@SiO_2_ composites with different mass ratios and different thickness are displayed in Figure S6. Therefore, it can be concluded that rGO/Fe_3_O_4_@SiO_2_, taking all the above aspects into account, may be applied practically as an outstanding absorber.

Moreover, the shielding effectiveness of the rGO/Fe_3_O_4_@SiO_2_ composite against electromagnetic interference (EMI) was evaluated based on the measured S parameters (S_11_, S_22_, S_12_, and S_21_). Transmission power (T) and reflection power (R) were obtained based on the expressions of T = |S_12_|^2^ = |S_21_|^2^, and R = |S_11_|^2^ = |S_22_|^2^, and absorption power (A) was calculated based on the expression A = 1 – R – T (Meng et al., 2014; Chen et al., 2015). The total EMI shielding effectiveness (SE) can be ascribed to contributions from reflection loss (SE_ref_), absorption loss (SE_abs_), and multiple reflections (SE_M_) (Song et al., 2014a; Zeng et al., 2015):

$$\begin{array}{l} SE\left(dB\right)=SE_{ref}+SE_{abs}+SE_{M}\\ \;SE_{ref}\left(dB\right)=10\log_{10}\left(1-R\right)\\ SE_{abs}\left(dB\right)=10\log_{10}\left[T/\left(1-R\right)\right] \end{array}$$

Usually, SE_M_ cannot operate effectively owing to the high frequency of measurement and the large distance between the interface and the reflector in comparison to the skin depth. The SE of materials can be formulated by using the primary data as follows (Kim et al., 2014; Song et al., 2014b; Mural et al., 2015):

$$\begin{array}{l} SE\left(dB\right)=10\times \log\left[\frac{1}{{\left|S_{12}\right|}^{2}}\right]=10\times \log\left[\frac{1}{{\left|S_{21}\right|}^{2}}\right] \end{array}$$

**Figure 4:**
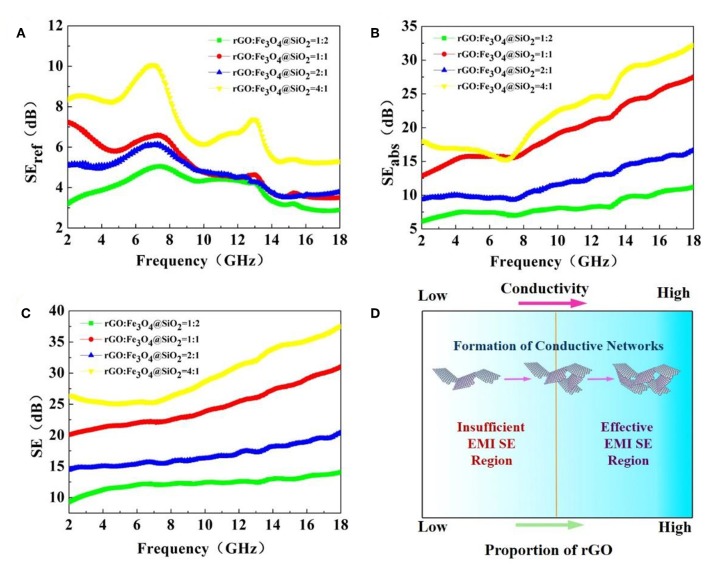
EMI shielding effectiveness in terms of the **(A)** SE_ref_, **(B)** SE_abs_, and **(C)** SE values of hybrids with various material proportions, and **(D)** illustration of two regions of EMI SE according to electrical conductivity and proportion of rGO.

It can be seen from Figure 4 that the values in the absorption part of their electromagnetic shielding performance are greater than those in the reflection part. The figure shows the change trend of the SE_ref_, SE_abs_, and SE values of the composite in the frequency range from 2 to 18 GHz as the proportion of rGO and Fe_3_O_4_@SiO_2_ changes. The Fe_3_O_4_@SiO_2_ nanochains, which is not composited with rGO, have weak electromagnetic shielding properties (shown in Figure S8). With an increasing proportion of rGO, the values of SE_ref_, SE_abs_, and SE are generally on the rise. Therefore, the result also remain basically consistent, which is the EM performance regulation of magnetic materials and conductive two-dimensional rGO is universal (Liu et al., 2016a,b, 2019; Xu et al., 2018b). Favorable electrical or magnetic conductivity is a necessary characteristic of shielding materials. Because an electromagnetic wave has both electric and magnetic field components, high conductivity is as important as permeability (Song et al., 2017). For high-frequency EMI (higher than 30 MHz), the conductivity of shielding materials is far more important than their permeability. Due to the excellent conductivity of rGO, the higher the proportion of rGO in composites, the better conductivity can be achieved, and, ultimately, conductive networks can be formed. The shielding effect is the best when the ratio is 4:1, with an average shielding efficiency of 30 dB and a maximum value of 37 dB at a frequency of 18 GHz, which indicates that it is a potential shielding material. Compared with other reported rGO-based nanofillers, rGO@Fe_3_O_4_@SiO_2_ in this study is a very competitive composites both in the fields of electromagnetic wave absorption and electromagnetic wave shielding (shown in Tables S1 and S2, respectively).

**Scheme 1:**
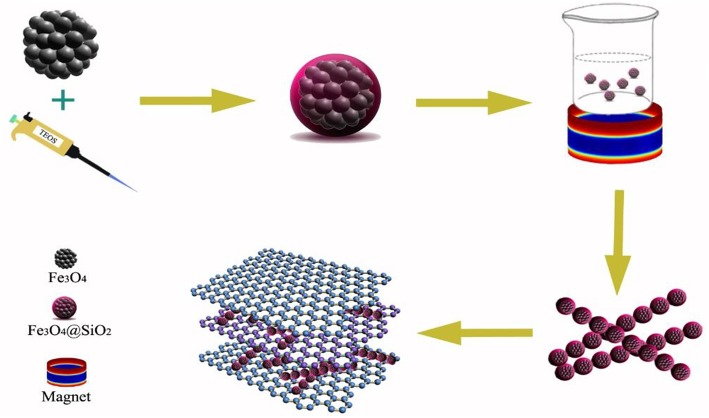
Simplified illustration of the procedure for rGO/Fe_3_O_4_@SiO_2_ composite synthesis.

## Conclusions

In this paper, Fe_3_O_4_@SiO_2_ nanochains were synthesized through the magnetic response of Fe_3_O_4_ MNCs and the hydrolysis of tetraethyl orthosilicate and were then coated with various proportions of rGO by adjusting the mass ratio. The composite showed excellent microwave absorption properties, with a maximum RL value of −48.34 dB and an effective absorption bandwidth of 4.88 GHz at a 2.5 wt% filler content. When the mass ratio of the composite is 4:1, the shielding effect is as desired, with an average shielding efficiency of 30 dB and a maximum value of 37 dB at the frequency of 18 GHz. The research results verify that this promising multi-functional hybrid can achieve considerable electromagnetic wave absorption performance or shielding performance at certain proportions of its constituent phases due to successful regulation of the dielectric constant and magnetic permeability.

## Data Availability Statement

The raw data supporting the conclusions of this manuscript will be made available by the authors, without undue reservation, to any qualified researcher.

## Author Contributions

C-QL, WX, and R-CD performed the main experimental operation and drafted the manuscript. XS and ZC performed the data analyses. M-DL and G-SW contributed to the conception of the study and financial support.

### Conflict of Interest

The authors declare that the research was conducted in the absence of any commercial or financial relationships that could be construed as a potential conflict of interest.
