# Supplementary material for: Tunable High-Performance Microwave Absorption and Shielding by Three Constituent Phases Between rGO and Fe3O4@SiO2 Nanochains
Source: Front Chem. 2019 Nov 26;7:711. doi: 10.3389/fchem.2019.00711 (PMC6901941; doi:10.3389/fchem.2019.00711)
Supplement: Supplementary file 1 [file Data_Sheet_1.docx]

Supplementary Material

Tunable High-performance Microwave Absorption and Shielding by Three Constituent Phases Between rGO and Fe_3_O_4_@SiO_2_ Nanochains

Chao-Qin Li ^1^, Wei Xu ^2^, Ruo-Cheng Ding ^2^, Xun Shen ^1^, Zhi Chen ^1^, Mao-Dong Li ^3*^, and Guang-Sheng Wang ^2*^

^1^Engineering Research Center of High-Performance Polymer and Molding Technology, Ministry of Education, Qingdao University of Science and Technology, Qingdao, PR China

^2^School of Chemistry, Beihang University, Beijing, PR China

^3^Guangzhou special pressure equipment testing and Research Institute, Guangzhou, PR China

*** Correspondence:**Guang-Sheng Wang
[wanggsh@buaa.edu.cn](mailto:wanggsh@buaa.edu.cn)


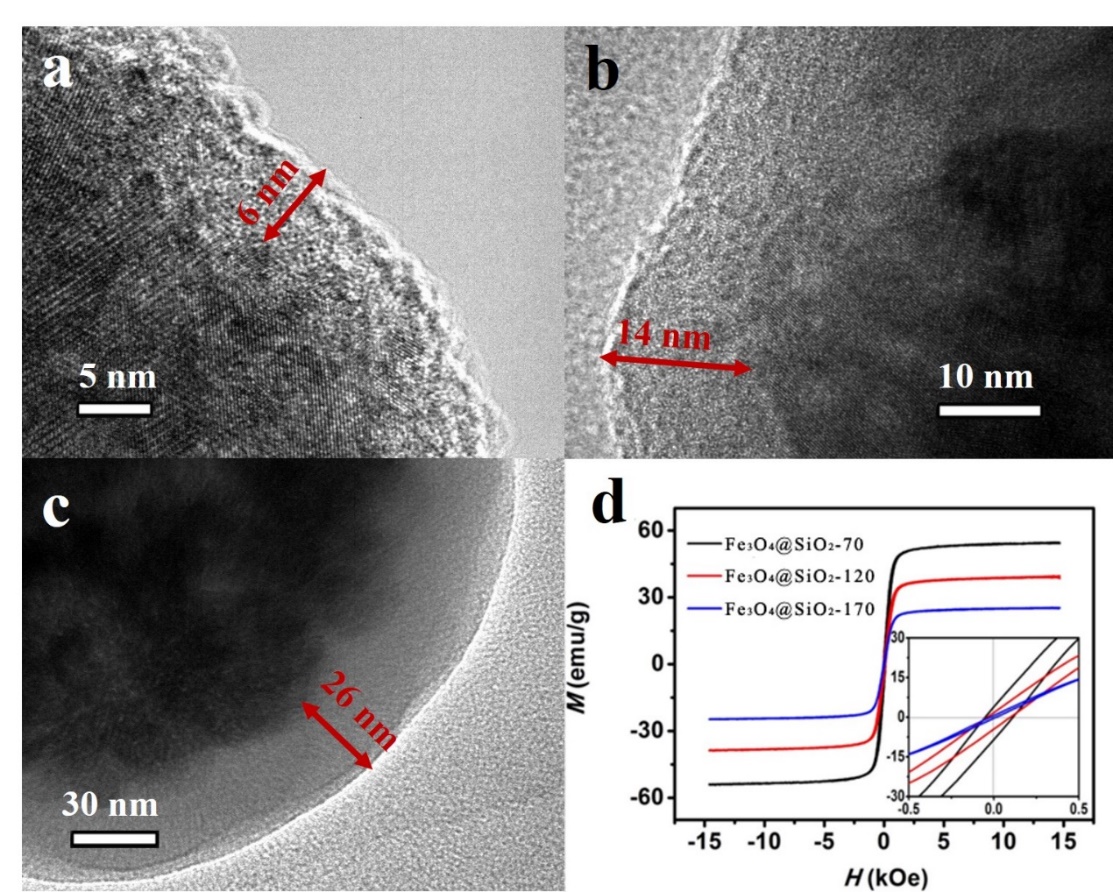


**Figure S1.** TEM images of Fe_3_O_4_@SiO_2_ nanochains for different amounts of TEOS: (a) 70 uL, (b) 120 uL, (c) 170 uL, respectively, and (d) magnetization hysteresis loops of samples measured at room temperature.


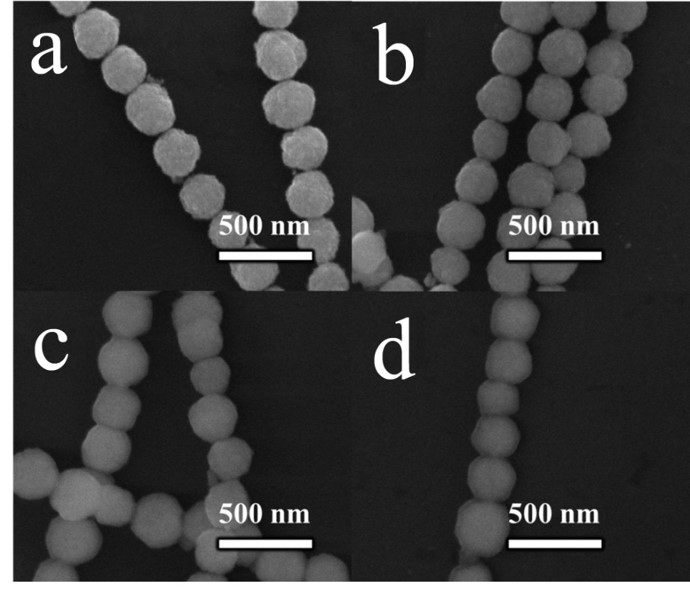


**Figure S2.** The samples were left to sit in a non-magnetic field (a) for 5 min, (b)for 10 min, (c) for 15 min, (d) for 20 min.


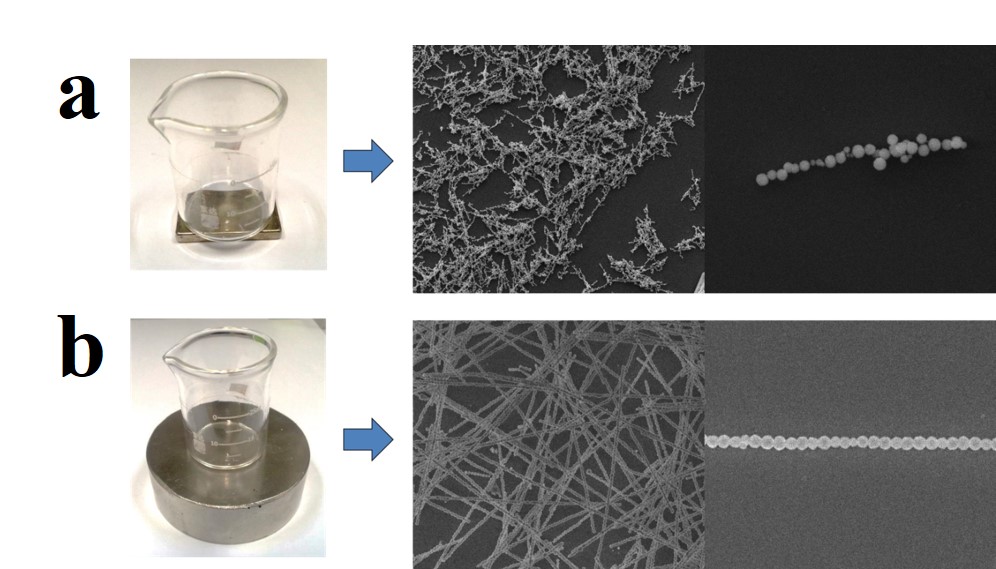


**Figure S3.** The effect of the uniformity of the applied magnetic field on the product of the Fe_3_O_4_/SiO_2_ nanochains: (a) the NdFeB magnets used were rectangular magnets of 40*25*5 mm and (b) disc magnets of 75*24 mm.


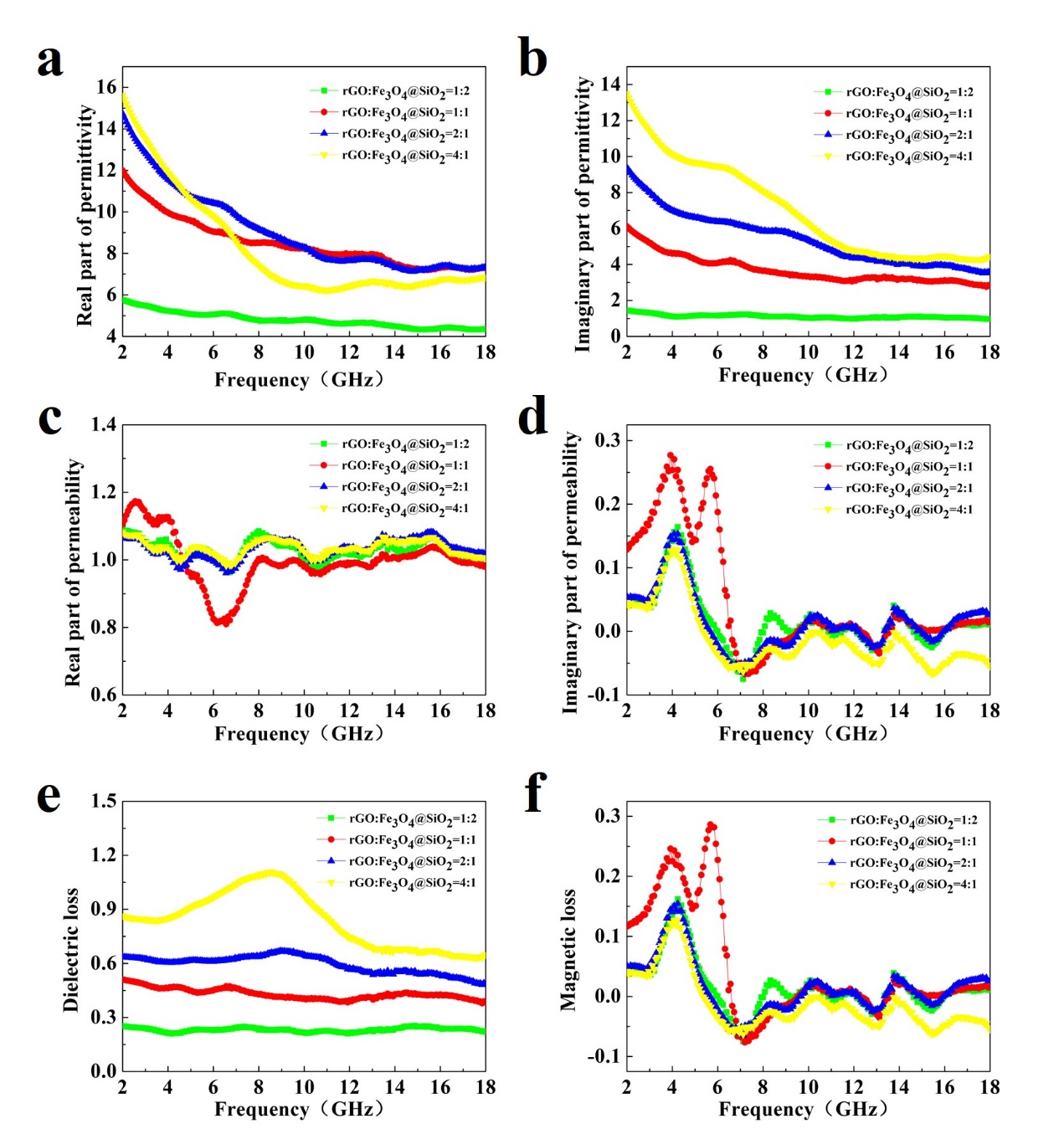


**Figure S4**. (a) Real and (b) imaginary parts of relative complex permittivity; (c) real and (d) imaginary parts of relative complex permeability; (e) dielectric loss and (f) magnetic loss for composites in the frequency range of 2–18 GHz.


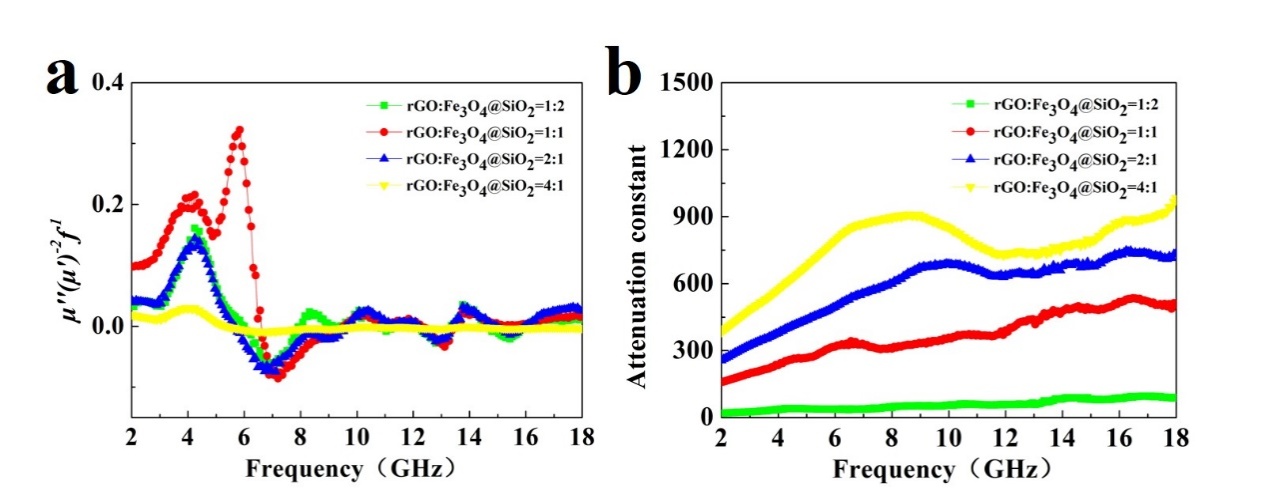


**Figure S5.** The *C_0_* – *f* curve (a) and attenuation constants (b) of rGO/Fe_3_O_4_@SiO_2_ with different mass ratios.


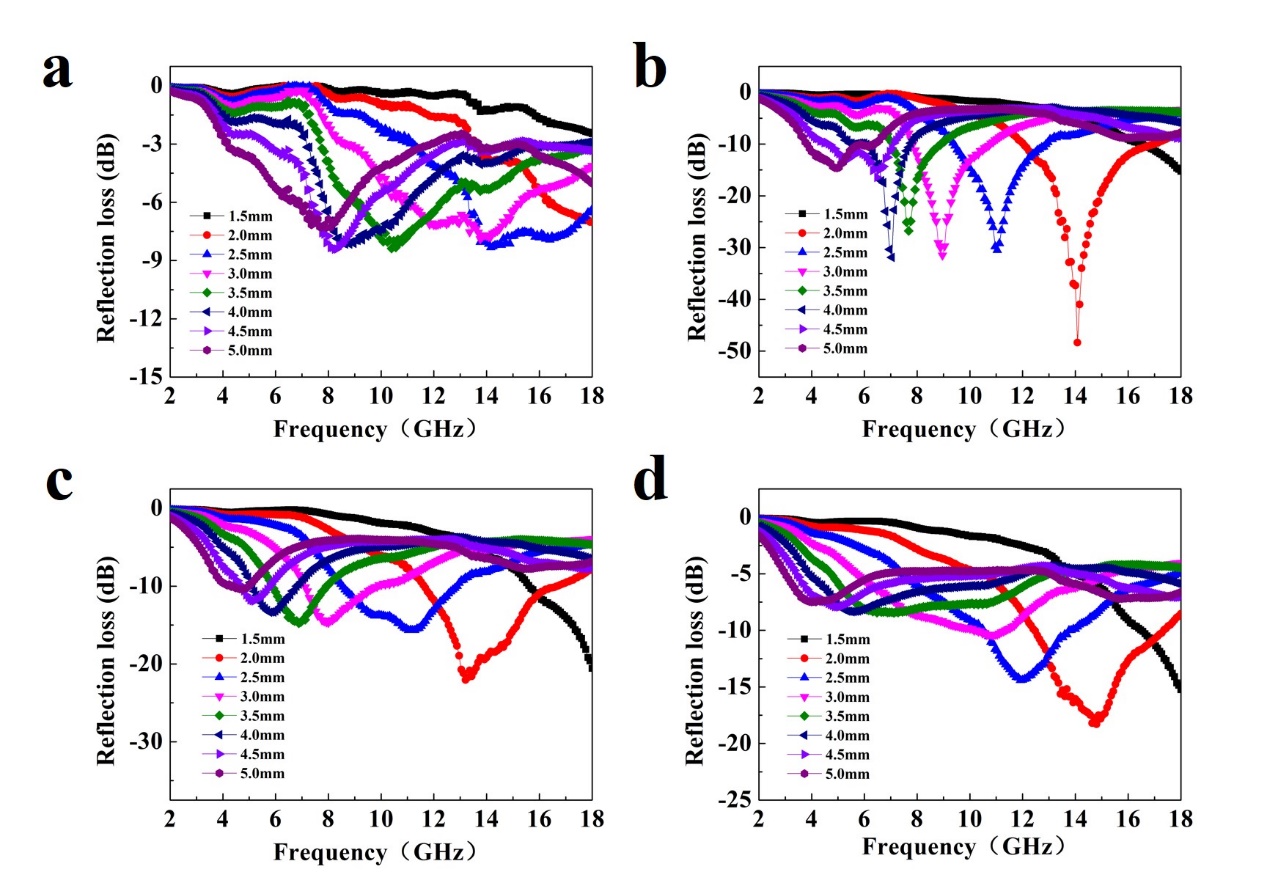


**Figure S6.** The microwave RL curves of rGO/Fe_3_O_4_@SiO_2_ composite with various thickness in (a) 1:2, (b) 1:1, (c) 2:1, (d) 4:1 mass ratio.


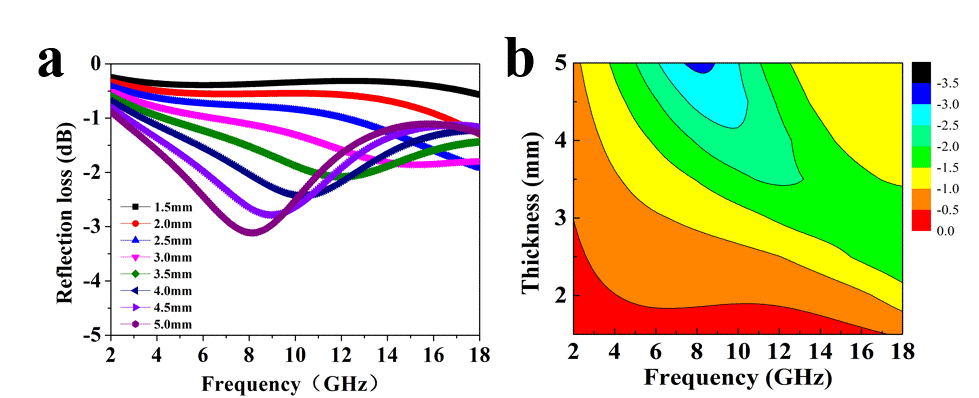


**Figure S7.** The microwave RL curves of (a) Fe_3_O_4_@SiO_2_ nanochains and (b) corresponding color fill versions.


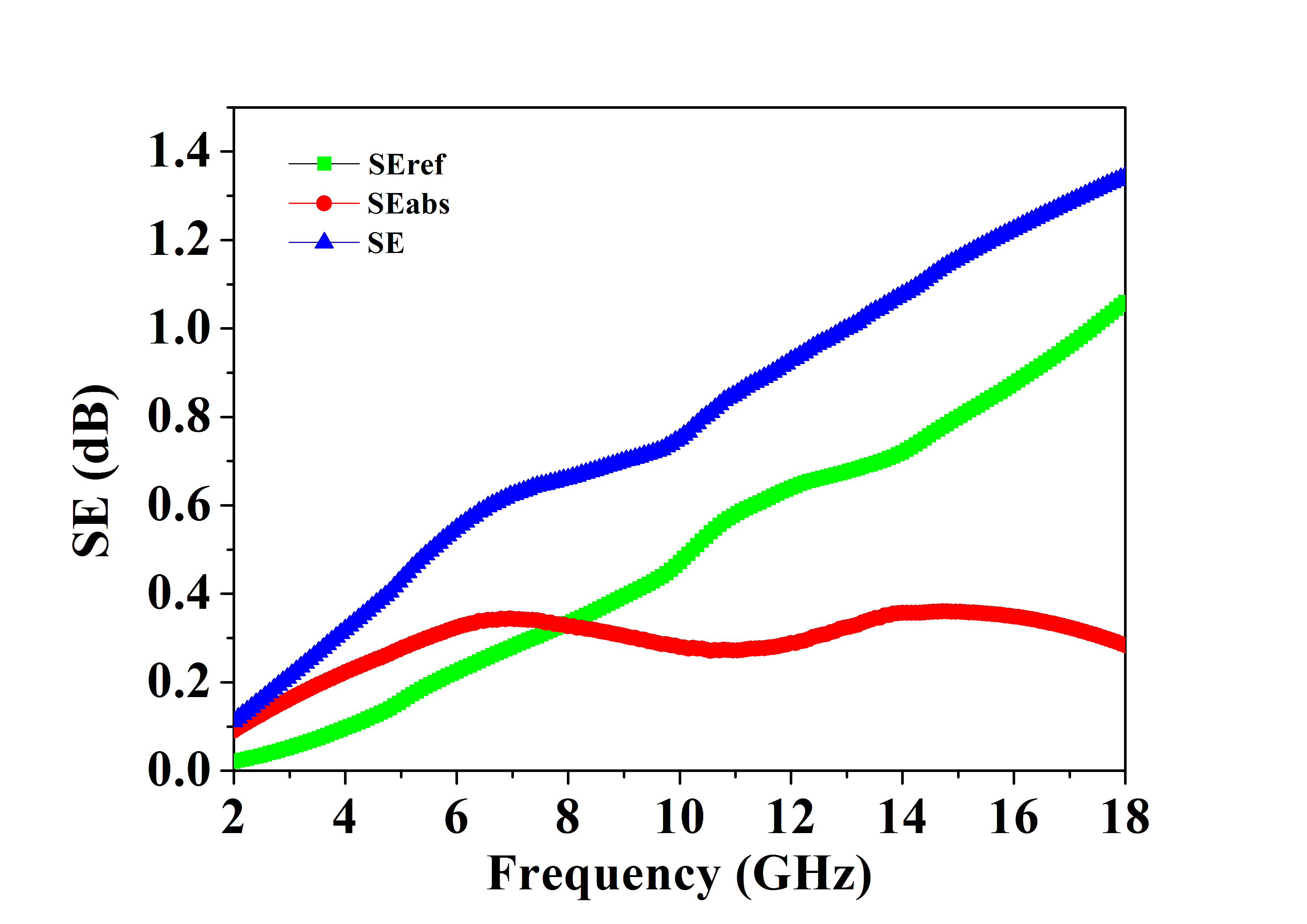


**Figure S8.** EMI shielding effectiveness of Fe_3_O_4_@SiO_2_ nanochains.

**Table S1.** The maximum RL values of different rGO-based nanofillers

| Filler | Filler content | Maximum RL value(dB) | References |
| --- | --- | --- | --- |
| rGO/MnFe_2_O_4_ | 5 wt% | -29.0 | [1] |
| rGO/Ni (nanochains) | 10 wt% | -39.0 | [2] |
| rGO/Fe_3_O_4_@SiO_2_ (nanospheres) | 20 wt% | -26.6 | [3] |
| rGO/Fe_3_O_4_@SiO_2_ (nanochains) | 2.5 wt% | -48.34 | This work |

**Table S2.** The maximum EMI SE values of different rGO-based nanofillers

| Filler | Filler content | Maximum EMI SE value(dB) | References |
| --- | --- | --- | --- |
| rGO/ Fe_3_O_4_ | 56 wt% | 11.2 | [4] |
| rGO/PS | 30 wt% | 29 | [5] |
| rGO@CB-Reinforced PDMS | 20 wt% | 28 | [6] |
| rGO/Fe_3_O_4_@SiO_2_ (nanochains) | 20 wt% | 37 | This work |

References

1. Zhang, X.-J., Wang, G.-S., Cao, W.-Q., Wei, Y.-Z., Liang, J.-F., Guo, L., et al. (2014). Enhanced microwave absorption property of reduced graphene oxide (RGO)-MnFe_2_O_4_ nanocomposites and polyvinylidene fluoride. *ACS Appl. Mater. Interfaces* 6, 7471-7478. doi: 10.1021/am500862g.
2. Xu, W., Wang, G.-S., and Yin, P.-G. (2018). Designed fabrication of reduced graphene oxides/Ni hybrids for effective electromagnetic absorption and shielding. *Carbon* 139, 759-767. doi: [10.1016/j.carbon.2018.07.044](https://doi.org/10.1016/j.carbon.2018.07.044).
3. Pan, Y.-F., Wang, G.-S., Yue, Y.-H. (2015). Fabrication of Fe_3_O_4_@SiO_2_@RGO nanocomposites and their excellent absorption properties with low filler content. *RSC Adv.* 5, 71718-71723. doi: 10.1039/c5ra13315g.
4. Yang, Y., Li, M., Wu, Y., Wang, T., Choo, E.S.G., Ding, J., et al. (2016). Nanoscaled self-alignment of Fe_3_O_4_ nanodiscs in ultrathin rGO films with engineered conductivity for electromagnetic interference shielding. *Nanoscale* 8, 15989-15998. doi: 10.1039/c6nr04539a.
5. Li, Y., Peng, Q., He, X., Hu, P., Wang, C., Shang, Y., et al. (2012). Synthesis and characterization of a new hierarchical reinforcement by chemically grafting graphene oxide onto carbon fibers. *J. Mater. Chem.* 22, 18748-18752. doi: 10.1039/c2jm32596a.
6. Anooja, J.B., Dijith, K.S., Surendran, K.P., and Subodh, G. (2019). A simple strategy for flexible electromagnetic interference shielding: Hybrid rGO@CB-Reinforced polydimethylsiloxane. *J. Alloys Compd.* 807, 151678. doi: <https://doi.org/10.1016/j.jallcom.2019.151678>.
